# A rare case of wandering spleen with splenomegaly in a postpartum female patient: A case report and literature review

**DOI:** 10.1016/j.ijscr.2024.110517

**Published:** 2024-10-24

**Authors:** Wongel Tena Shale, Tilahun Habte Nureta, Ahmed Yusuf Omar, Godfrey Sama Philipo

**Affiliations:** a Jimma University Institute of Health, Department of Surgery, P.O. Box 378, Jimma, Oromia, Ethiopia; b Jimma University Institute of Health, Department of Surgery, Gastrointestinal Surgery Unit, P.O. Box 378, Jimma, Oromia, Ethiopia; c College of Surgeons of East, Central, and Southern Africa, 157 Olorien, Njiro Road ECSA-HC, P.O.Box 1009, Arusha, Tanzania

**Keywords:** Wandering spleen, Hypermobile spleen, Postpartum, Splenectomy

## Abstract

**Background:**

Wandering spleen or hypermobile spleen results from the elongation or maldevelopment of the spleen’s suspensory ligaments. Few cases have been reported worldwide, making it a rare clinical entity. It usually affects children, although it also commonly affects female adults in the reproductive age range.

**Case presentation:**

A 30-year-old para 2 female patient presented with left upper quadrant abdominal pain of 3 days duration. She gave birth to a living baby 4 months before her current presentation. Her abdomen was distended, and there was a palpable mass on the central abdomen area. CT showed absence of spleen in the splenic fossa anterior to the left kidney, a wandering spleen with no attachment, and a whirl sign. The patient was managed with splenectomy and autotransplantation.

**Discussion:**

Wandering spleen is one of the rare surgical conditions. The majority of affected females are multiparous, and women between the ages of 20 and 40 are approximately 13 times more likely than men to present with this condition. Ligamentous laxity of the spleen can be caused by physiologic splenomegaly and hormonal changes during pregnancy.

**Conclusion:**

Wandering spleen associated with splenomegaly is a rare clinical condition. These patients require a cautious approach due to the hypermobile spleen's propensity for torsion and infarction.

## Highlights

• Wandering spleen is a rare clinical condition with an incidence of 0.2%
• Most commonly affected demographics are children and women between ages 20 and 40 years
• Its frequent correlation with pregnancy and postpartum is said to be caused by hormonal fluctuations and physiological changes
• Treatment is almost always surgical. Splenectomy is the preferred in case of splenic pedicle torsion and subsequent infarction.

## 1 Introduction

Wandering spleen is a rare clinical entity that results from the absence or maldevelopment of the ligaments that support the spleen in its normal location [1]. As a result, the spleen is hypermobile and may be predisposed to hilar torsion and subsequently infarction, making it a potentially fatal emergency. It is an uncommonly encountered condition that mainly affects the pediatric population in a third of cases [2]. In adults, females of reproductive age group are mostly affected, with the cause hypothesized to be hormonal changes during pregnancy leading to ligamentous laxity [1,2]. It can present as an asymptomatic, palpable abdominal mass or with acute, chronic, or intermittent symptoms due to torsion of the wandering spleen [1,2]. Due to rarity and various modes of presentation, it has proven difficult for the clinician to diagnose and treat it. Here we report a case of a 30-year-old multiparous woman who presented with acute abdomen due to a wandering spleen complicated with splenomegaly, torsion, and splenic infarction. This case report is reported according to the Surgical Cases Report (SCARE) guideline [9].

## 2 Case presentation

A 30-year-old female patient presented with abdominal pain of 3 days duration. The pain was dull aching type which was more pronounced over the left upper quadrant, and it was aggravated by movements like changing position, and it was associated with increasing abdominal grith and loss of appetite but no vomiting. The patient was from malaria-endemic area and she was diagnosed and treated for malaria a year back. She is a para 2 mother with her last delivery being 4 months before her presentation, and both deliveries were spontaneous vaginal deliveries in a health center. She has no previous history of surgery or chronic medical conditions.

On presentation, she looked acutely sick with pain. Her blood pressure was 108/70 mmHg, her pulse rate was 102 beats per minute and she was afebrile. Her abdomen was distended with a palpable mass on the left upper quadrant measuring 15cmx 10 cm with smooth margins and firm in consistency extending up to the midline with localized tenderness and guarding. Examining fingers could go between the mass and the costal margin. A digital rectal exam revealed normal findings.

**Fig. 1:**
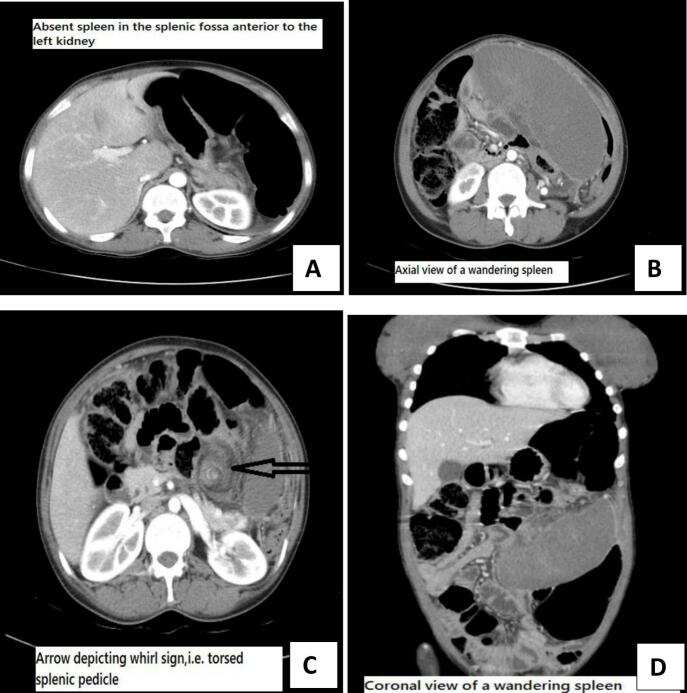
An axial view showing absent spleen in the splenic fossa anterior to the left kidney(A), Wandering enlarged spleen with no attachments to the posterior abdominal wall(B), Whirlpool sign (arrow) depicting torsed splenic pedicle(C), Coronal view of CECT depicting wandering spleen.

Laboratory parameters were as follows. Her hemoglobin was 11 g/dL and her white blood cell count was 7.54 × 10^3^/mm^3^. The platelet count was normal. Liver function and renal function tests were within the normal range. Abdominal ultrasound showed empty splenic fossa and anterior abdominal mass likely wandering spleen with lower pole infarction. Contrast enhancing abdominal CT scan (CECT) was done to confirm the diagnosis and it showed the absence of spleen in the splenic fossa anterior to the left kidney, wandering spleen with no attachment to the abdominal wall, and whirl sign depicting torsed splenic pedicle (Fig. 1).

### 2.1 Management

The patient was taken to the operating room for exploratory laparotomy.

### 2.2 Intraoperative findings

**Fig. 2:**
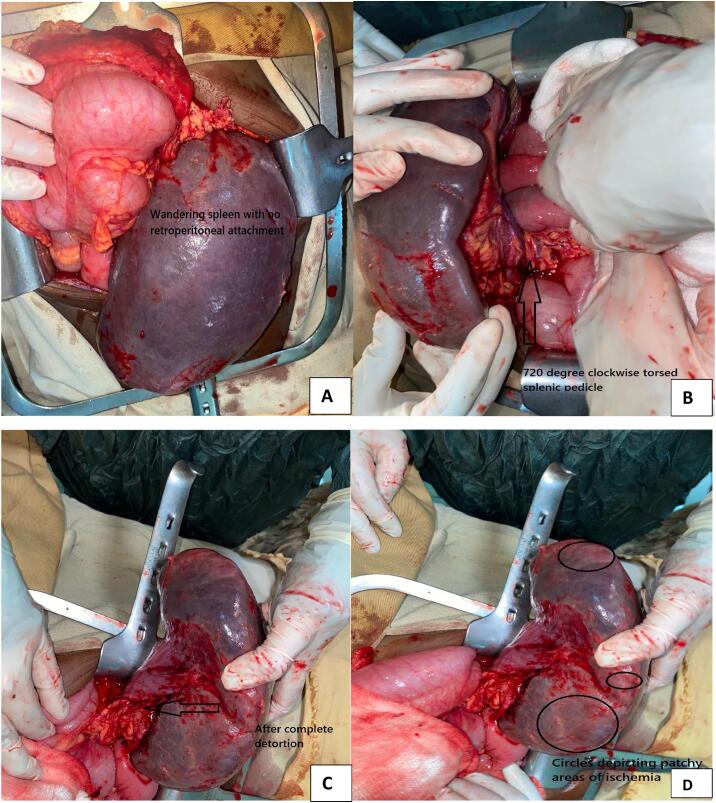
An intraoperative image of an enlarged wandering spleen with no peritoneal attachment to the posterior abdominal wall (A), splenic pedicle twisted 720 degrees clockwise (B), splenic pedicle after complete detorsion, arrow (C), patchy ischemic areas marked by circles.

There was an enlarged spleen measuring 15 cm × 12 cm with no peritoneal attachment to the posterior abdominal wall. The spleen was located in the central abdomen and left upper quadrant. Patchy ischemia was evident on the anterior surface and it was adherent to the small bowel and greater omentum draping over it. Additionally, the splenic pedicle was twisted 720° (Fig. 2).

### 2.3 Done

The adhesion was gently released and total splenectomy was done. Autotransplantation of the splenic tissue was also done.

The patient had a smooth post-operative course and was discharged on the 4th postoperative day. She was prescribed post-splenectomy vaccination upon referral. She was followed for 1 year after the surgery and her recovery was uneventful.

**Fig. 3:**
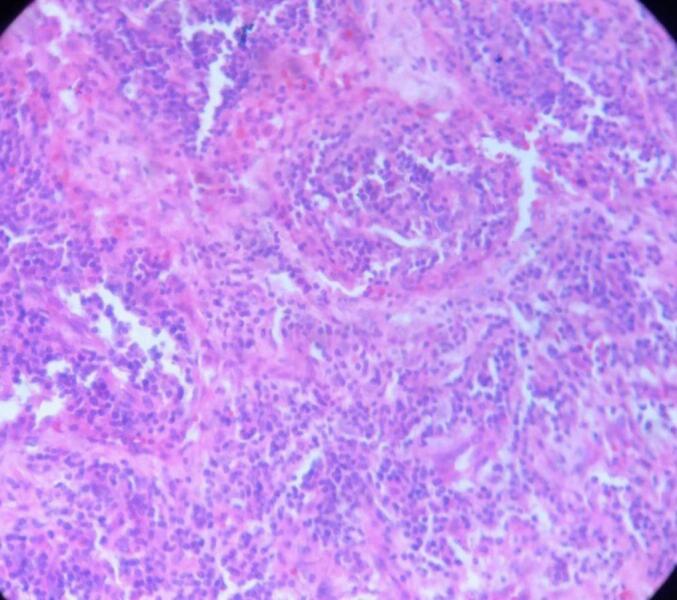
Normal appearing splenic parenchyma along with extensive hemorrhage and coagulative necrosis.

Histopathology: The splenic specimen was sent for histopathological analysis. The result showed normal-appearing splenic parenchyma along with extensive hemorrhage and coagulative necrosis, with findings consistent with infarcted spleen (Fig. 3).

## 3 Discussion

Wandering spleen, also known as the hypermobile spleen, refers to the abnormal increase in mobility of the spleen so that it migrates from its normal position in the left hypochondriac fossa because of the excessively long splenic pedicle and improper fixation of the splenic tissue to the underlying posterior abdominal wall [3]. The first case of wandering spleen was reported by Von Horne in 1667. Wandering spleen is found in less than 0.2 % of splenectomies and occurs mainly in children and women aged 20–40 years [4,7]. Less than 600 cases of wandering spleen have been reported to date [4].

The splenic ligaments are formed by the condensation of the peritoneum and serve to anchor the spleen into the posterior abdominal wall. These are splenorenal, gastrosplenic, splenocolic, and splenophrenic ligaments. Abnormality or absence of these ligaments can lead the spleen to be abnormally hypermobile [5]. Congenitally, the wandering spleen is caused by these ligaments failing to form, which leads to a lengthy splenic mesentery. The spleen grows in the dorsal mesogastrium and shifts posterolateral to the left when the gut rotates. The lienorenal ligament, which houses the tail of the pancreas and the splenic artery, is formed by the fusion of the dorsal mesogastrium with the posterior abdominal wall and the left kidney. Failure of fusion produces an abnormally long pedicle [6]. Acquired cases appear mainly associated with parity, which causes weakness of the abdominal wall and laxity of ligaments normally attached to the spleen. It may also be associated with splenomegaly [4]. This occurs frequently in multiparous women, such as our patient, who gave birth four months before her presentation. In addition to that the patient also came from a malaria-endemic area, and had a history of repeated malaria attacks, which might have contributed to the splenomegaly, further adding to her risk.

Women are about 13-fold more prone to this entity than men, the age at presentation maybe 20 to 40 years, and most affected females are multiparous [1]. There are only 12 documented cases of wandering spleen either during pregnancy or the postpartum period [20,21]. Ten of them have available abstracts in English (Table 1). All the documented cases were managed with splenectomy, and the majority had good outcomes. Two of them experienced spontaneous abortion. The presence of long splenic pedicles, which carry the blood supply to the spleen, predisposes to torsion of the pedicle leading to a partial or complete splenic infarction. In addition, the risk of torsion is aggravated if the spleen weighs greater than 500 g [4].

**Table 1:** List of cases of wandering spleen during pregnancy and postpartum till July 2024.

Authors	Age	Time of diagnosis	Clinical presentation and intraoperative finding	Management
Rasheed O. et al., 2024 [10]	34	postpartum	Abdominal pain and distention History of splenic vein thrombosis	Splenectomy, after delivery
Anyfantakis D. et al., 2013 [11]	19	Postpartum (10 days)	Abdominal pain, diffuse abdominal tenderness and guarding Splenic pedicle torsion	Total splenectomy
Gilman RS et al., 2003 [12]	24	Pregnancy (36 weeks)	Acute pancreatitis, thrombocytopenia, Upper gastrointestinal bleeding	Splenectomy
Chekol, Alemneh Mitku et al., 2024 [13]	30	Pregnancy (12 weeks+5)	Abdominal pain and tenderness Splenic pedicle torsion and infarction, splenomegaly	Splenectomy
Lewis DL et al., 1962 [14]	28	Pregnancy (4 months)	Abdominal pain, tenderness, nausea, vomiting, small bowel obstruction, splenic torsion, spontaneous abortion of twin pregnancy	Splenectomy
Lahiri, Somdatta et al., 2010 [15]	18	Pregnancy (5 weeks)	Acute abdomen, shock, splenic torsion, splenic rupture, spontaneous abortion	Splenectomy
Yücel, Ergün et al., 2012 [16]	24	Pregnancy (18 weeks)	Intraabdominal mass and lower abdominal pain	Laparoscopic splenectomy
Ghazeeri, Ghina et al., 2010 [17]	23	Pregnancy (12 weeks)	Abdominal pain, tenderness, intractable vomiting, left lower quadrant mass, thrombocytopenia	
Parvaiz A. et al., 2004 [18]	27	Pregnancy (36 weeks)	Tachycardia, shock, hemoperitoneum, infarcted and lacerated spleen	Splenectomy
Ravid, A et al., 1999 [19]	26	Pregnancy	Abdominal pain, thrombocytopenia, Splenic torsion	Splenectomy

The clinical presentation is variable. The majority of them may be asymptomatic. However, some require surgical intervention due to acute or chronic torsion, which can result in symptoms including abdominal discomfort, a mass in the abdomen, symptoms of splenic infarction, or left side portal hypertension [1]. The most typical manifestation is a mass accompanied by vague abdominal symptoms or sporadic abdominal pain brought on by congested torsion and spontaneous detorsion [4]. In our case, it was acute abdominal pain for 3 days with no prior history.

The clinical diagnosis of a wandering spleen can be challenging, but the diagnosis can readily be made by cross-sectional imaging investigations. A computed tomography scan of the abdomen can demonstrate the absence of a spleen in its normal position in the left upper quadrant of the abdomen along with the presence of a mass in the lower abdominopelvic cavity that has similar attenuation to that of the normal splenic tissue [7].

Surgical treatment is the only recommended management for wandering spleen [4]. Splenectomy is often required in cases of splenic torsion with infarction. It may also be advised in the massive wandering spleen that is not amenable to splenopexy. In other situations, splenic preservation with splenopexy should be attempted to avoid the future risks of overwhelming post-splenectomy sepsis. Surgical treatment is needed even in asymptomatic patients because the rate of complications is substantial [4]. Autotransplantation was additionally done for our patient by taking a tiny portion of the excised spleen and sectioned into thin, small pieces which were reimplanted to a heterotopic location in the peritoneal cavity (the greater omentum). We often practice autotransplantation when we perform splenectomies for trauma or when the splenic parenchyma is not significantly diseased. Evidence shows that the practice improved post-splenectomy outcomes by preserving some of the splenic functions [22]. Even though there is evidence showing splenic autotransplantation could preserve some function of the spleen. No adequate data confirms the decreased occurrence of overwhelming post-splenectomy Infection (OPSI). Therefore, we prescribe vaccination against capsulated microorganisms despite autotransplantation.

Conservative management of wandering spleen is not advised, as it is associated with a complication rate that exceeds 50 %.

## 4 Conclusion

Wandering spleen associated with splenomegaly is a rare clinical condition, but the most frequently affected population is pediatrics and women in their childbearing age. These patients require a cautious approach due to the hypermobile spleen's propensity for torsion and infarction, which can result in an acute abdomen. When there is splenic infraction and concomitant splenomegaly, splenic preservation surgery is not an option. Although it is possible to auto-transplant the spleen, its long-term effects are yet unknown.

## Consent

The patient gave their written, informed consent in the original language for the release of any non-identifying information, including any photos taken during the procedure. The journal's Editor-in-Chief may evaluate a copy of the written consent upon request.

## Ethical approval

Ethical approval is exempt by the IRB of Jimma University for case reports as long as written consent is obtained from the patient, patient identifying information is removed, and the study doesn't involve human or animal experiments.

## Funding

No funding was used for this case report.

## Declaration of competing interest

No conflict of interest to declare.
